# A survey-based study of knowledge of Alzheimer’s disease among health care staff

**DOI:** 10.1186/1471-2318-13-2

**Published:** 2013-01-02

**Authors:** Wendy Smyth, Elaine Fielding, Elizabeth Beattie, Anne Gardner, Wendy Moyle, Sara Franklin, Sonia Hines, Margaret MacAndrew

**Affiliations:** 1 Tropical Health Research Unit, The Townsville Hospital and Health Service, Internal mail box 105, The Townsville Hospital, PO Box 670, Townsville, QLD, 4810, Australia; 2 Dementia Collaborative Research Centre - Carers & Consumers, School of Nursing, Queensland University of Technology, Brisbane, QLD, Australia; 3 School of Nursing, Midwifery & Paramedicine, Australian Catholic University, PO Box 256, Dickson, ACT 2602, Australia; 4 School of Nursing, Midwifery & Nutrition, James Cook University, Townsville, QLD, Australia; 5 Griffith Health Institute, Griffith University, Brisbane, QLD, Australia; 6 Nursing Research Centre, Mater Health Services, Brisbane, QLD, Australia

**Keywords:** Aged care, Alzheimer’s disease, Dementia, Knowledge, Health care staff

## Abstract

**Background:**

Continued aging of the population is expected to be accompanied by substantial increases in the number of people with dementia and in the number of health care staff required to care for them. Adequate knowledge about dementia among health care staff is important to the quality of care delivered to this vulnerable population. The purpose of this study was to assess knowledge about dementia across a range of health care staff in a regional health service district.

**Methods:**

Knowledge levels were investigated via the validated 30-item Alzheimer’s Disease Knowledge Scale (ADKS). All health service district staff with e-mail access were invited to participate in an online survey. Knowledge levels were compared across demographic categories, professional groups, and by whether the respondent had any professional or personal experience caring for someone with dementia. The effect of dementia-specific training or education on knowledge level was also evaluated.

**Results:**

A diverse staff group (N = 360), in terms of age, professional group (nursing, medicine, allied health, support staff) and work setting from a regional health service in Queensland, Australia responded. Overall knowledge about Alzheimer’s disease was of a generally moderate level with significant differences being observed by professional group and whether the respondent had any professional or personal experience caring for someone with dementia. Knowledge was lower for some of the specific content domains of the ADKS, especially those that were more medically-oriented, such as ‘risk factors’ and ‘course of the disease.’ Knowledge was higher for those who had experienced dementia-specific training, such as attendance at a series of relevant workshops.

**Conclusions:**

Specific deficits in dementia knowledge were identified among Australian health care staff, and the results suggest dementia-specific training might improve knowledge. As one piece of an overall plan to improve health care delivery to people with dementia, this research supports the role of introducing systematic dementia-specific education or training.

## Background

Internationally the provision of effective and appropriate care for people with dementia is a growing challenge. In Australia, it is estimated that in 2011, nearly 300,000 people had dementia out of a total population of 23 million. This number is projected to increase to 900,000 by 2050, with a parallel increase required in the number of health professionals to care for this vulnerable population [1]. The quality of life as well as the functional status of people with dementia is affected by the quality of their care in a variety of health care environments, including acute and community care. Adequate knowledge of dementia among health care staff has been shown to affect critical issues in care, such as the timing of diagnosis and the subsequent implementation of interventions and the quality of care environments [2-4], which are in turn, linked to improved patient outcomes [3,5,6]. However, previous studies have shown that those responsible for the diagnosis of dementia, the implementation of treatment plans, and the daily care of people with dementia have deficits in dementia knowledge.

Both positive and negative outcomes of the level of dementia knowledge on the part of health professionals, family caregivers and the people with dementia themselves have been highlighted in previous research. When these groups possess a good level of knowledge about dementia, it has been found that: diagnosis will occur at an earlier stage as the person with dementia will seek medical advice earlier; family members may raise an alert earlier and health professionals will recognise the symptoms of early dementia [3,6-8]; the person with dementia is given more opportunity and choices regarding disease management plans [4,7,9,10]; and the incidence of stigma associated with a diagnosis of dementia may be reduced [7]. On the other hand, poor knowledge about dementia has been found to result in the underutilisation of support and treatment services [11] and in delayed diagnosis which prolongs the initiation of treatment plans and results in poorer outcomes for people with dementia and their caregivers such as inadequate care of the disease, misinterpretation of behaviours and increased caregiver stress due to failure to seek appropriate support [7,11]. Primary care physicians (general practitioners or GPs in Australia) receive minimal specific training in geriatrics during their undergraduate studies, and both GPs and nurses have self-reported that they have a knowledge deficit about dementia [2,3,6]. Similarly, certified nursing assistants are exposed to the least amount of formal knowledge regarding dementia during their training, yet are responsible for the provision of up to 90% of the basic care for people with dementia [12]. Assessing and addressing this knowledge deficit may be one of the keys to improving dementia care and health outcomes in the future.

In the early stages of dementia, many people first present with symptoms to a GP or to a health care worker in an acute hospital when seeking treatment for a seemingly unrelated condition [2]. However, these doctors and nurses, when not gerontological specialists, have been shown to be generally lacking in dementia knowledge [7]. In the acute care setting, with 15 – 50% of patients having a degree of cognitive impairment [13], knowledge of dementia among those involved in their care, including medical practitioners, allied health staff, social workers, clerical staff and nurses, is essential. Even cleaning, maintenance and security staff in acute care environments come into contact with people with cognitive impairments and might benefit from greater general knowledge about dementia. For example, acute care nurses without a good understanding of dementia have admitted to: having great difficulty interpreting behaviours of dementia; prioritising care to those without dementia as it would take too long to deal with the person with dementia who is unable to communicate; and to experiencing fear of caring for patients with dementia because they believe they are at risk of harm [14]. Other research has demonstrated that quality care of the person with dementia is feasible in the acute care setting, when attention to the special needs of patients with dementia is given [15].

Two recent studies conducted in Australia corroborate these points about the relationship between knowledge of dementia and quality of care in the acute care environment [13,16]. Partly because of lack of knowledge about dementia and the possible sources of patient confusion, acute care of cognitively impaired people was found to be inconsistent and to have emphasised safety at the expense of wellbeing and dignity [16]. Care staff displayed generally negative attitudes towards people with dementia [16] and nurses expressed reluctance to care for patients with dementia because they found it unrewarding [13]. Both of these sets of authors concluded that staff education and training was critical to improving the quality of care received by a person with dementia while in acute care [13,16]. The first step toward improving dementia knowledge of all types of health care staff is to assess their current level of dementia knowledge using a reliable tool that will identify knowledge gaps.

There are a number of dementia knowledge assessment tools that have been developed over the past decades. A recent review [7] of such tools concluded that while the Alzheimer’s Disease Knowledge Test (ADKT) [17] was the oldest and most widely used, a more recent tool, the Alzheimer’s Disease Knowledge Scale (ADKS) [18], was promising and showed good psychometric properties. Originally only used by its developers [19], two recently published articles have confirmed the utility of the ADKS in this context [20,21]. Assessing the level of dementia knowledge among health professionals is important to: assess the level of knowledge prior to the implementation of dementia knowledge programs as a baseline; identify the gaps in knowledge to be included in a program; and then to assess the effectiveness of a dementia knowledge education program [2,6,7,12,22]. This study aims to ascertain staff knowledge about dementia across a regional health service region in Queensland, Australia using the ADKS.

Two primary research questions guided this study. These were:

1. What knowledge of dementia do health care staff (nursing, medical, allied health, and support) in a large regional health district have?

2. Controlling for demographic characteristics, how are dementia-specific education or training and experience caring for someone with dementia related to knowledge about dementia?

## Methods

A cross-sectional survey design was used. Human research ethics approval was obtained for the project as a low risk study from the health service region and one participating university.

### Measures

The recently developed Alzheimer's Disease Knowledge Scale (ADKS) [18] was selected to gauge knowledge, because of its ease of use, demonstrated reliability and validity, and applicability for different groups of participants (general public, caregivers, and health professionals). The ADKS is an updated version of the Alzheimer's Disease Knowledge Test [17] and consists of 30 true/false items with the resulting score being the number answered correctly [18]. Reliability of the ADKS measured by test-retest correlation = .81; internal consistency, as measured by the average inter-item correlation of α = .71 and content/predictive validity is adequate. The ADKS is conceptually split into the following seven content domains: *life impact* (items 1, 11 and 28), *risk factors* (items 2, 13, 18, 25, 26 and 27), *symptoms* (items 19, 22, 23 and 30), *treatment and management* (items 9, 12, 24 and 29), *assessment and diagnosis* (items 4, 10, 20 and 21 ), *care giving* (items 5, 6, 7, 15 and 16) and *course of the disease* (items 3, 8, 14 and 17) [18].

Respondents in the current study were also asked to rate their own level of knowledge about dementia on an 11-point Likert scale (0 = *I know nothing at all* to 10 = *I am very knowledgeable*). Two questions gauged respondents’ experience of caring for people with dementia: one focussed on non-work-related or personal caring and another specifically asking about professional (or work-related) caring. Demographic questions on the survey assessed: age group, family history of dementia, professional group (nursing, medicine, allied health or support), work setting (hospital versus community) and geographic location within the district (inside or outside the major regional city). In the area studied, the professional group classifications listed would be understood as follows: nursing (registered and enrolled nurses, assistants in nursing), medicine (general medical practitioners, specialists), allied health (other professionals employed by the health department including speech, physical, occupational and recreational therapists; social workers; and psychologists), and support (administrative, cleaning, patient transport and security). To measure dementia-specific training or education, respondents could choose any one or more of seven types, which were grouped into three categories as follows: 1) “tertiary education” included those who had taken an undergraduate or postgraduate course with explicit dementia content; 2) “dementia training” represented those having attended a dementia-specific conference, a hospital in-service dementia course, or a workshop or session run by one of two dementia-specific training organisations in the state (Alzheimer’s Australia-Queensland or the Eastern Australia Dementia Training Study Centre) and 3) “Other dementia learning” represented those declaring self-directed (e.g. from online content or reading) or “other” learning in the area of dementia. Survey questions other than the ADKS were pre-tested by staff at the regional hospital and the research institutions involved.

### Population and sample

The chosen health service region in Queensland (Australia) covers 750,000 square kilometres with one tertiary referral level acute hospital, several small regional health facilities and community health services. In Australia, the provision of public health services is the responsibility of each state government, which divides the state into regions (or districts) in order to manage service provision, as well as the referral process. The population of the area is approximately 160,000, with a high proportion being of northern European ancestry. A new Acute Delirium/Dementia Unit is located in the regional tertiary referral level hospital. During the study period the health service district employed 4,753 staff, most of whom had employer-provided e-mail access. The sampling frame comprised all staff with e-mail access.

### Process

The survey was conducted online using SurveyMonkey™ software. Mass e-mails containing a link to the survey were sent to all staff in the health district, using the internal email system. In order to maximise response rate, a modified Dillman [23] process was used, involving two main strategies: 1) pre-advertising the survey via electronic and paper posters distributed to all health service delivery sites in the region and 2) an initial email describing the study followed by two reminder emails, all with a link to the online survey. The initial call for participants was distributed during Dementia Awareness Week in September 2009, immediately after the opening of a new Acute Delirium/Dementia Unit at the regional hospital. In total, the survey was accessible for approximately four weeks.

### Respondents

Of the approximately 4,750 regional health service staff with email accounts, 1,659 opened the survey email, and 410 people commenced the survey. Of the 410 people starting the survey, 360 respondents completed the main dependent variable scale (Alzheimer’s Disease Knowledge Scale) and their responses comprised the final data set. This final sample represented a response rate of 21.7% (360/1659), with the denominator being the people who received the survey as defined by having opened the email.

### Data analysis

Data analysis was carried out using IBM SPSS Statistics 19™. Two tests were undertaken to check for potential biases in the sample achieved. The first was comparing the demographic characteristics of the respondents completing the ADKS with those not completing it. Because no statistically significant differences were found, no corrections were made. The second potential bias was whether the sample accurately represented the population of all regional health service staff. When the demographic characteristics of the respondents were compared to those of the population (data privileged), several statistically significant differences were found (at the .05 level). The sample of respondents contained relatively more females, fewer people under 30 years of age (and more people over 50 years old), and more allied health staff (and fewer medical staff) than the population. Because of these differences, a population weighting procedure was employed to adjust for resulting biases in the results. Weights were computed to conform to the marginal percentages of the gender, age, and professional group distributions in the population simultaneously, using an iterative, raking procedure [24]. Weighted and unweighted results were compared; given that significant differences were found, weighted results are shown in all tables.

Descriptive statistics for all potential independent variables (weighted mean for self-knowledge and weighted percentages for all others) were calculated. The status of each independent variable as a predictor was evaluated at the bivariate level through comparing means on the ADKS measure with ANOVA tests. Linear regression analysis was used to determine the independent impact of these variables on ADKS score. Because of strong associations between the professional caring experience variable and the three education/training variables (χ^2 ^values all less than .001), a choice needed to be made as to which to include in the regression models in order to avoid the impact of multicollinearity. Because the primary substantive interest was in the potential effect of dementia-specific education and training, these variables were tested in the regression analyses (and the professional experience variable was not).

## Results

The respondents were diverse in terms of age, professional group, and work setting. Most respondents worked in the central regional city area and most were female, as expected given the workforce in the health service district [25]. Dementia had affected the personal lives of many of the respondents; approximately 40% reported having non-work-related (personal experience) caring for someone with dementia and 30% had a family history of dementia (Table 1). Additionally, 62% of the respondents had cared for someone with dementia in the workplace (professional experience). Many of the respondents reported some dementia-specific preparation in the form of university education (31%), training (21%) or other learning (41%) (Table 1).

**Table 1:** Background characteristics and their relationship to dementia knowledge (N = 360)

**Background characteristics**	**Unweighted number (Weighted **%**)**	**ADKS**	**F**	**df**	**Significance**
		**Mean (SD)**			
**Demographics**					
**Gender (n = 356)**					
Male	55 (21.5)	23.53 (3.30)	0.03	1,353	.854
Female	301 (78.5)	23.61 (3.21)			
**Age**					
<30 years	41 (17.3)	24.67 (2.85)	5.01	2,356	.007
30-50 years	185 (51.7)	23.49 (3.22)			
>50 years	134 (31.0)	23.07 (3.43)			
**Professional Group**					
Nursing	169 (46.2)	23.90 (2.84)	17.32	3,355	<.001
Medicine	11 (10.3)	26.0 8 (1.80)			
Allied Health	81 (11.8)	23.87 (3.69)			
Support	99 (31.7)	22.14 (3.41)			
**Work setting (n = 355)**					
Community	100 (24.6)	24.02 (3.44)	2.42	1,353	.121
Hospital	255 (74.2)	23.40 (3.18)			
**Geographic Location**					
Urban Centre	311 (87.6)	23.56 (3.26)	0.000	1,357	.986
Rural Area	49 (12.4)	23.56 (3.00)			
**Experience and education**					
**Family history of dementia (n = 359)**					
Yes	106 (29.4)	23.91 (3.26)	1.94	1,357	.165
No	253 (70.6)	23.43 (3.26)			
**Personal caring experience**					
Yes	149 (41.4)	23.88 (3.23)	3.89	1,358	.049
No	211 (58.6)	23.34 (3.28)			
**Professional caring experience**					
Yes	222 (62.2)	24.38 (2.97)	40.85	1,358	<.001
No	138 (37.8)	22.22 (3.41)			
**Tertiary Education**					
Yes	92 (31.0)	24.72 (3.09)	21.58	1,358	<.001
No	268 (69.0)	23.04 (3.21)			
**Dementia training**					
Yes	87 (20.7)	24.32 (3.10)	5.06	1,358	.025
No	273 (79.3)	23.37 (3.28)			
**Other dementia learning**					
Yes	152 (41.4)	24.06 (2.95)	5.93	1,358	.015
No	208 (58.6)	23.21 (3.43)			
**Self rated dementia knowledge (n = 325)**		5.15 (2.23)	r = .367		<.001

The overall mean score of dementia knowledge as measured by the ADKS was 23.6 (SD = 3.26) out of 30 (79% correct). The following variables displayed a statistically significant relationship with ADKS score: age, professional group, personal caring experience, professional caring experience, tertiary education, dementia training, and other dementia learning. In terms of specific categories of respondents for the multi-category variables, those less than 30 years old and those in the medical professional group scored best on the ADKS, while support staff displayed significantly lower scores.

In rating their own knowledge of dementia on the 11-point scale, health district employees reported on average a moderate knowledge of dementia (mean = 5.2, SD = 2.23; Table 1). Respondents who rated themselves as having more knowledge of dementia tended to score significantly higher on the ADKS (r = .37, p < .001).

As shown in Table 2, respondents’ scores (in terms of percent correct out of total possible) on most of the seven domains within the ADKS were similar and in the range 80–87 percent. Two domains stood out as having lower scores—*risk factors* (percent correct = 65%) and *course of the disease* (75%). These two domains contained the most medically oriented questions, such as those about what factors predispose people to developing dementia and how long the course of the disease typically lasts. Professional group was related to the knowledge of dementia in specific content domains, with the relationship being statistically significant in five of the seven content domains. Respondents with medical training scored particularly high in the *risk factors* domain compared to the other three groups, while scores for support staff were the lowest among all groups across all domains.

**Table 2:** ADKS content domains and professional group

				**Professional group**						
				**Nursing**	**Medical**	**Allied health**	**Support**			
**Domain**	**# items**	**Mean/SD**	**% Correct**	**Mean/SD**	**Mean/SD**	**Mean/SD**	**Mean/SD**	**F**	**DF**	**Sig**
**Life Impact**	3	2.60 (0.58)	87%	2.63 (0.57)	2.93 (0.25)	2.62 (0.52)	2.44 (0.63)	7.79	3,355	<0.001
**Risk Factors**	6	3.90 (1.22)	65%	3.92 (1.14)	4.81 (1.19)	3.87 (1.08)	3.58 (1.24)	10.24	3,355	<0.001
**Symptoms**	4	3.18 (0.88)	80%	3.20 (0.85)	3.65 (0.49)	3.12 (0.94)	3.01 (0.94)	5.12	3,355	0.002
**Treatment management**	4	3.42 (0.74)	86%	3.45 (0.72)	3.53 (0.51)	3.47 (0.82)	3.30 (0.81)	1.30	3,355	0.275
**Assessment**	4	3.42 (0.78)	86%	3.49 (0.76)	3.75 (0.61)	3.49 (0.72)	3.17 (0.82)	6.89	3,355	<0.001
**Care giving**	5	4.05 (0.90)	81%	4.16 (0.84)	4.16 (0.76)	4.25 (0.89)	3.79 (0.97)	5.07	3,355	0.002
**Course of the disease**	4	3.00 (0.91)	75%	3.05 (0.87)	3.26 (0.77)	3.05 (0.90)	2.84 (1.00)	2.33	3,355	0.075

A linear regression analysis predicting ADKS score including all demographic variables yielded the parsimonious result (in which only significant predictors are included) shown in Model 1 of Table 3. Being the largest category and in the absence of a substantive reason for choosing among professional groups, nursing was used as the omitted category in the regression. Age and professional group were independent predictors, with those less than 30 scoring about 1 point higher, those in the medicine group scoring 1.8 points higher and those in the support group 1.8 point lower on the ADKS (other things equal). This model explained about 12% of the variance in ADKS score. For the second model, personal caring experience and the three education/training variables were added with only personal experience and dementia-specific training emerging as additional significant predictors of ADKS (Model 2, Table 3). Those with personal experience caring for someone with dementia had a predicted 0.7 point higher score on the ADKS compared with those without experience, and those who had undergone formal dementia-specific training showed a predicted 0.8 point higher score than those without such training. Professional group remained an independently significant predictor, while age was no longer important in this model.

**Table 3:** Regression models predicting knowledge of dementia

	**Model 1**			**Model 2**				
**Variables**	**b (SE)**	**t**	**sig**	**b (SE)**	**t**	**sig**		
**Constant**	23.62 (.330)	71.51	<.001	23.39 (.293)	79.95	<.001
**Age**						
Less than 30	1.03 (.511)	2.02	.044			
30-50	.341 (.366)	0.93	.352			
More than 50 (omitted)						
**Professional Group**						
**Nursing (omitted)**						
Medical	1.81 (.591)	3.07	.002	2.38 (.558)	4.27	<.001
Allied health	−0.18 (.531)	−0.34	.732	−.075 (.521)	−0.14	.886
Support	−1.81 (.372)	−4.86	<.001	−1.64 (.377)	−4.35	<.001
**Personal caring experience**				0.69 (.325)	2.12	.035
**Dementia training**				0.84 (.422)	2.07	.039
**Adjusted R**^**2**^	0.125			0.136		
**N**	359			359		

## Discussion

The findings from this diverse group of health district staff show a generally moderate level (average of 79% correct) of dementia knowledge. As expected, those in professions with direct patient contact (medical, nursing and allied health) showed higher levels of knowledge than those in a supportive role (administrative, housekeeping, security and transport staff). There were, however, deficits across almost all respondents in specific areas of the assessment, namely in the domains *risk factors* and *course of the disease*. In terms of overall results on the chosen measure of dementia knowledge, these results closely resembled the results from the original study [18,19]. The mean score on the ADKS in the current sample of health district staff (M = 23.6) was similar to that for dementia caregivers (M = 22.7) and older adults (M = 24.1) in the original study. In addition, the current study confirmed positive correlations between self-assessed knowledge of dementia and having attended a dementia-specific educational session with ADKS score observed in the original study [19].

This study may be limited by the fact that respondents were a self-selected group and it is possible that those who tended to respond were those with an interest in, or knowledge about, dementia. The ADKS is focused on knowledge of Alzheimer’s disease specifically and therefore does not evaluate knowledge of the other dementias. Furthermore, there was a considerable amount of variance in knowledge of dementia which cannot be explained by either the demographic characteristics or experience caring for, or learning specifically about caring for, people with dementia. However, the variance explained in this study (14%) is notably higher than the percent (8%) in the one other study found predicting ADKS with demographic and professional characteristics [20]. Because the current study was conducted in only one health service region in Australia, it is difficult to know how closely the sample reflects the wider community, both in Australia and elsewhere.

## Conclusions

The significance of these results can be placed in the context of which elements might be under the control of health departments. One of the important factors shown by this research to predict knowledge of dementia, personal experience caring for someone with dementia, is not externally modifiable. However, two aspects of these results are significant to potentially improving the publicly provided health care of people with dementia: 1) highlighting which specific areas of dementia knowledge are often lacking and 2) demonstrating that having dementia-specific training and/or education is associated with greater general dementia knowledge, independent of demographic and personal experience. Understanding the risk factors for and course of Alzheimer’s disease (both lacking in the current sample) is generally considered core knowledge underpinning quality dementia care. Knowledge of risk factors is also important from the perspective of general population health and staff members’ personal health behaviours and risk profiles. On the other hand, one of the strongest independent predictors of dementia knowledge, education or training in the area, can be controlled by employers and encouraged in health degree programs.

Dementia education has been identified as a means of improving dementia knowledge especially for health professionals [9]. In recent studies, several dementia education programs and resources were tested for their effectiveness to improve knowledge of dementia. These included: an information video and written information developed by Alzheimer’s Australia [22]; a pamphlet developed by Alzheimer’s Australia (‘Mind Your Mind’) [6]; a one hour lecture covering the information tested in the knowledge of dementia measure [11]; a six hour course covering the physiology of dementia, common behaviours seen in dementia and management of behaviours such as wandering, confusion and communication difficulties [12]; and education on dementia integrated within an overall demonstration program to improve dementia care and management [13]. With the exception of the one hour lecture [11], all the education programs and resources studied were found to improve knowledge of dementia when assessed using the various tools available. In particular, Foreman and Gardner (2005) found that the education and training program improved attitudes towards caring for people with dementia as well as general knowledge of dementia among health care staff [13]. In confirming the link between dementia-specific training and knowledge of dementia, this study adds to the existing literature and has implications for both care and policy regarding acute and community care of people with dementia. Consequently, if we are to truly support quality dementia care, then one goal for health services could be to achieve a high proportion of staff who have undergone dementia-specific education. This may require that health service regions include dementia education as a mandatory component of both employment criteria and staff development programs for staff working and caring for older people. Any such education or training would need to target all staff who would potentially have contact with patients with dementia. Support staff cannot normally be expected to have had the health background or opportunity to learn specifically about this vulnerable population and their unique needs. The projected growth in numbers of older people with dementia in acute settings suggests that staff from all levels will come into contact with this group at some stage; therefore appropriate education and training will help in the delivery of quality dementia care.

It needs to be noted that knowledge alone does not necessarily translate into change in care [26]. Conversely, high quality care is not solely dependent on broad education about dementia; but our results suggest that dementia-specific education could be an important contributor to change. Staff involved in direct patient care will need a comprehensive extended education program, with information about degrees of severity of dementia and which provides participants with specific skill sets to enable the delivery of high quality care of these patients in an acute setting. One possible area in need of further research is to examine the specific types or elements of dementia education or training that are associated with better attitudes towards, and confidence with, the care of patients with dementia. One possible study would be a pre- and post-design to evaluate changes in knowledge of dementia, and attitudes towards caring for people with dementia, among health professionals who receive an educational intervention.

## Competing interests

The authors declare that they have no competing interests.

## Authors’ contributions

WS assisted in designing the study, preparing the ethics, selecting survey tools, distributing the survey, reviewing the findings, reviewing the literature, and drafting the manuscript. EF was instrumental in undertaking the data analysis and interpreting the findings, and contributed to drafting the manuscript. EB guided the research team, conceptualised the study, and was involved in all phases of the study and drafting the manuscript. AG assisted in designing the study, preparing the ethics, reviewing the findings, reviewing the literature and drafts of the manuscript. WM assisted in designing the study, reviewing the findings, reviewing the literature and drafts of the manuscript. SF primarily assisted in data analysis and interpretation, and in drafting the manuscript. SH assisted in designing the study, reviewing the literature, selecting survey tools, and drafting the manuscript. MM primarily assisted in reviewing the literature, drafting and formatting the article. All authors read and approved the final manuscript.

## Authors’ information

WS: PhD, RN; EF: PhD; EB: PhD, RN; AG: PhD, RN; WM: PhD, RN; SF: BPsych(Hons); SH: MAppSci(Research), RN; MM: BAppSc, RN

## Pre-publication history

The pre-publication history for this paper can be accessed here:

http://www.biomedcentral.com/1471-2318/13/2/prepub
